# High expression of cellular retinol binding protein-1 in lung adenocarcinoma is associated with poor prognosis

**DOI:** 10.18632/genesandcancer.89

**Published:** 2015-11

**Authors:** Elena Doldo, Gaetana Costanza, Amedeo Ferlosio, Eugenio Pompeo, Sara Agostinelli, Guido Bellezza, Donatella Mazzaglia, Alessandro Giunta, Angelo Sidoni, Augusto Orlandi

**Affiliations:** ^1^ Anatomic Pathology, Department of Biomedicine and Prevention, Tor Vergata University of Rome, Italy; ^2^ Thoracic Surgery, Tor Vergata Policlinic of Rome, Italy; ^3^ Department of Experimental Medicine, Section of Anatomic Pathology and Histology, Medical School, University of Perugia, Italy; ^4^ Department of Anatomic Pathology, Tor Vergata Policlinic of Rome, Italy

**Keywords:** lung cancer, CRBP-1, Akt, Erk, EGFR, prognostic marker, survival

## Abstract

**Purpose:**

Adenocarcinoma, the most common non-small cell lung cancer is a leading cause of death worldwide, with a low overall survival (OS) despite increasing attempts to achieve an early diagnosis and accomplish surgical and multimodality treatment strategies. Cellular retinol binding protein-1 (CRBP-1) regulates retinol bioavailability and cell differentiation, but its role in lung cancerogenesis remains uncertain.

**Experimental design:**

CRBP-1 expression, clinical outcome and other prognostic factors were investigated in 167 lung adenocarcinoma patients. CRBP-1 expression was evaluated by immunohistochemistry of tissue microarray sections, gene copy number analysis and tumor methylation specific PCR. Effects of CRBP-1 expression on proliferation/apoptosis gene array, protein and transcripts were investigated in transfected A549 lung adenocarcinoma cells.

**Results:**

CRBP-1^High^ expression was observed in 62.3% of adenocarcinomas and correlated with increased tumor grade and reduced OS as an independent prognostic factor. CRBP-1 gene copy gain also associated with tumor CRBP-1^High^ status and dedifferentiation. CRBP-1-transfected (CRBP-1^+^) A549 grew more than CRBP-1^−^ A549 cells. At >1μM concentrations, *all trans*-retinoic acid and retinol reduced viability more in CRBP-1^+^ than in CRBP-1^−^ A549 cells. CRBP-1^+^ A549 cells showed up-regulated RARα/ RXRα and proliferative and transcriptional genes including pAkt, pEGFR, pErk1/2, creb1 and c-jun, whereas RARβ and p53 were strongly down-regulated; pAkt/pErk/ pEGFR inhibitors counteracted proliferative advantage and increased RARα/RXRα, c-jun and CD44 expression in CRBP-1^+^ A549 cells.

**Conclusion:**

CRBP-1^High^ expression in lung adenocarcinoma correlated with increased tumor grade and reduced OS, likely through increased Akt/Erk/EGFR-mediated cell proliferation and differentiation. CRBP-1^High^ expression can be considered an additional marker of poor prognosis in lung adenocarcinoma patients.

## INTRODUCTION

Non-small-cell lung cancer (NSCLC) is a leading cause of cancer death worldwide [1]. Lung cancer is divided in two major categories according to histological features and response to conventional therapies. More than 85% of lung malignant tumors are NSCLC [2] and adenocarcinoma frequence largely prevails among lung NSCLCs hystotypes [3]. Despite the recent advances in diagnostic and therapeutic procedures, including the development of computed tomography-based screening programs for early detection of lung cancer in higher risk populations, overall survival (OS) in lung adenocarcinoma patients remains poor with a heterogeneous and as yet suboptimal response rates to both surgical and multimodality chemo-radiation therapeutic approaches [3]. Research effort has been focused on identifying new biomarkers and those molecular pathways influencing critically NSCLC progression. The discovery of activating EGFR mutations and the subsequent development of tyrosine kinase inhibitors led to a revolution in the treatment of NSCLC patients [4]. Recently, the screening of other prognostic genetic factors or biomarkers influencing DNA repair mechanisms and inflammatory response have been suggested to predict recurrence or metastasis of NSCLC [5]. The discovery of additional markers of NSCLC heterogeneity remains a goal to suggest new therapeutic perspectives and clinical trials to improve individual therapeutic response. Vitamin A (retinol) and its metabolites are essential for many biological processes and influence epithelial cell differentiation and proliferation [6]. Biological activity of retinol is normally mediated by specific receptors. Retinol mediates pleiotropic and transcriptional effects of retinoids through the binding to nuclear receptors, namely the retinoic acid receptors (RARα, β, and γ) and retinoid X receptors (RXRα, β, and γ) [7]. Recently, increased interest has been focused on the role of cellular retinol and retinoic acid binding proteins (CRBPs and CRABPs) in carcinogenesis. Physiologically, CRBPs and CRABPs regulate intracellular retinoid trafficking and retinoid-induced cell activities. CRBP-1 is a 15 kDa cytosolic binding protein crucial for the uptake and subsequent esterification of retinol, so regulating of its bioavailability and transcriptional activities [6] [8]. CRBP-1 is indispensable for embryonic development, growth, vision and survival of vertebrates and in lung transient CRBP-1 expression is reported during pre-natal alveolus formation [9]. A potential role of CRBP-1-driven aberrant intracellular retinoid signaling in non-lung cancer carcinogenesis has been highlighted [10-13]. Natural and synthetic retinoids are effective for the treatment of skin proliferative disorders and also represent chemopreventive agents [14,15]. Therapeutic employment of retinoids in NSCLC patients gave controversial results and a link to smoking habits suggested [16]. Here, we aimed to define if CRBP-1 expression can influence tumor progression and OS in lung adenocarcinoma patients. CRBP-1 expression and its relationship with survival and other prognostic factors was investigated in a series of 167 adenocarcinoma patients. The effects of CRBP-1 transfection on proliferation, transcription, dedifferentiation and sensitivity to retinoids in A549 adenocarcinoma cells were also reported and discussed.

## RESULTS

### CRBP-1 expression and clinicopathological characteristics

**Figure 1:**
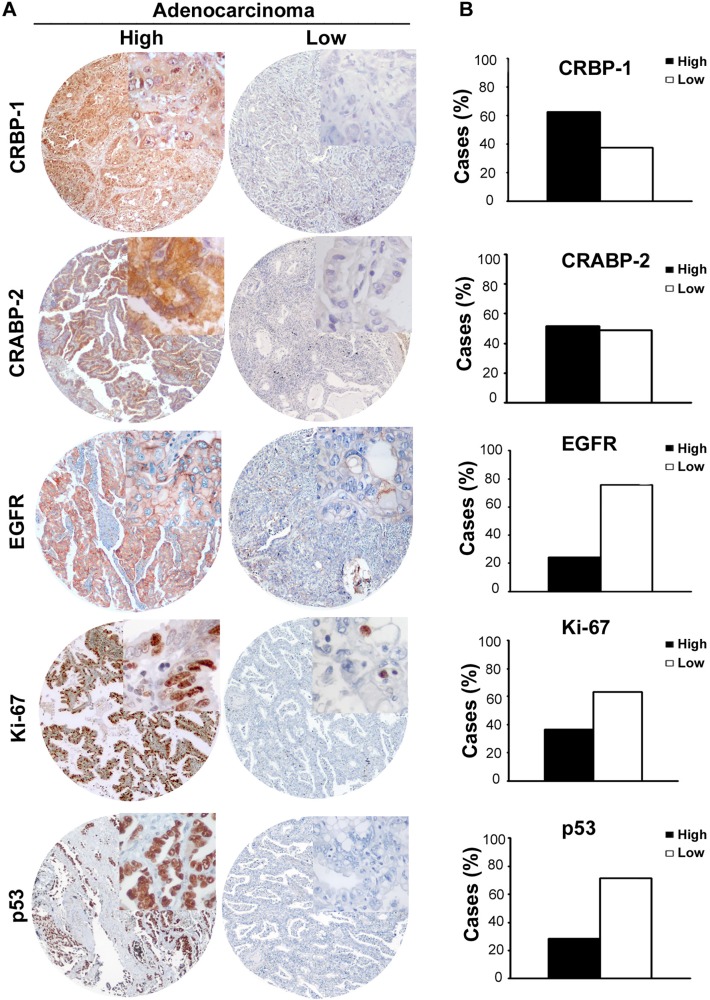
Immunostaining and semiquantitative evaluation of CRBP-1 and other biomarkers in TMA sections of lung adenocarcinomas A, representative images of “High” and “Low” CRBP-1, CRABP-2, EGFR, Ki-67 and p53 expression in lung adenocarcinoma. B, bar graphs showing the percentages of tumors with “High” and “Low” expression. Diaminobenzidine is used as chromogen. Criteria for the definition of “High” and “Low” tumors are reported in the Patients and Methods section. Original magnification, 40X; the inset highlighting the staining at higher magnification, 400X.

Representative immunostainings of CRBP-1 expression and other lung adenocarcinoma biomarkers are reported in figure 1A. As shown in figure 1B, CRBP-1^High^ expression was detected in 62.3%. EGFR and Ki-67 expression were comparable to the literature [17,18]. CRABP-2^High^ expression was detected in 51.85% of tumors. Immunohistochemistry also documented that CRBP-1 expression was absent in non-neoplastic lung parenchyma and present in bronchial epithelium (Supplementary Figure S1A-F). CRBP-1 expression varied according to the hystopathological subtype (Supplementary Figure S1G). In particular, acinar, micropapillary, solid and mucinous subtypes resulted more frequently CRBP-1^High^, whereas CRBP-1^Low^ expression prevailed among lepidic and papillary subtypes. Patients' clinicopathological characteristics and their relationship with CRBP-1 expression are reported in table 1. Smokers status (current or former smokers) showed a significant association with tumor CRBP-1^High^ expression (p<0.001). As concerning the tumor grade, CRBP-1^High^ status associated to the loss of differentiation of lung adenocarcinomas (p<0.001).

**Table 1:** Clinicopathologic characteristics by CRBP-1 expression and factors influencing survival in lung adenocarcinoma patients

	Total Number	CRBP-1 expression		
		Low (%)	High (%)	
Variable *	167 (%)	63 (37.7)	104 (62.3)	*P*
Sex				
male	128 (76.6)	48	80	0.91
female	39 (23.4)	15	24	
Age				
< 67 years	85 (50.9)	32	53	0.98
> 67 years	82 (49.1)	31	51	
Smoking status				
current or former smokers	138 (82.6)	40	98	0.001
no-smokers	29 (17.4)	23	6	
Histologic grade				
G1	18 (10.8)	13	5	0.001
G2+G3	149 (89.2)	50	99	
pT				
T1+T2	134 (80.2)	49	85	0.37
T3+T4	33 (19.8)	14	19	
Disease stage				
I	112 (65.6)	43	69	0.37
II+III+IV	55 (34.4)	20	35	

Variable **	*P*	HR^a^	95% Cl^b^
CRBP-1	0.02	0.43	0.21-0.89
EGFR	0.001	3.18	0.67-6.05
CRABP-2	0.13	1.61	0.86-3.00
Ki-67	0.3	0.72	0.38-1.33
RARβ	0.09	0.58	0.31-1.08
RARα	0.68	0.87	0.45-1.69
Smokers/CRBP-1High	0.07	0.50	0.64-1.06
Coexpression EGFR/CRBP-1	0.24	0.56	0.21-1.46

* χ2 test was used for relationships;

** Multivariate Cox Regression Model;

a Hazard Ratio;

b Confidence Interval

### CRBP-1^High^ expression in lung adenocarcinoma associates with reduced overall survival

**Figure 2:**
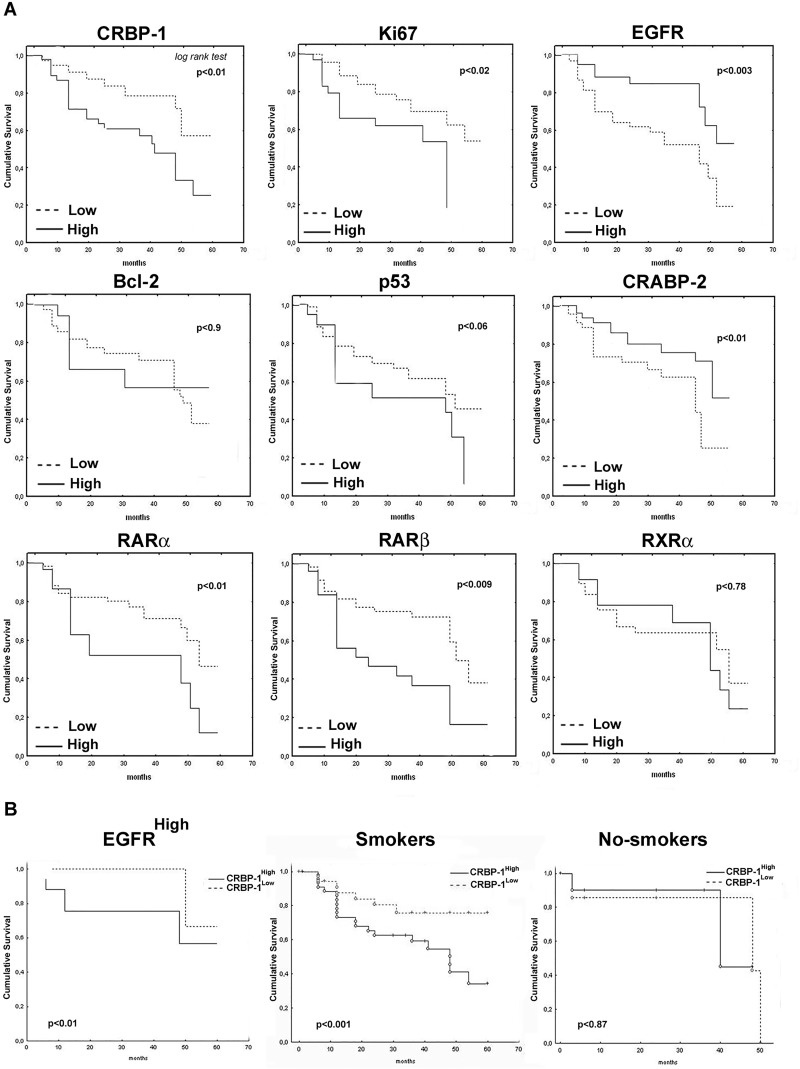
Overall survival and CRBP-1 and other biomarker expression in lung adenocarcinoma patients A, CRBP-1 and other biomarker expression and overall survival in lung adenocarcinoma patients (n=167). B, survival rate of patients with tumor CRBP-1^High^ and EGFR^High^ coexpression and smoking habits in lung adenocarcinoma patients. Significance is calculated by *Log rank test*.

As reported in figure 2A, CRBP-1^High^ expression in lung adenocarcinoma associated with reduced patients' OS (p<0.01). High RARα, RARβ and Ki-67 (p<0.01; p<0.009 and p<0.02, respectively) and low CRABP-2 and EGFR expression (p<0.01 and p<0.003, respectively) also correlated with lower OS, similarly to that reported in the literature [19]. As reported in figure 2B, tumor subgroup with EGFR^High^ and CRBP-1^High^ coexpression associated with reduced OS (p<0.01). CRBP-1^High^ expression strongly also associated with reduced OS in smoker (p<0.001) but not in no-smoker patients. Multivariate analysis (Table 1) documented CRBP-1^High^ and EGFR^High^ expression as independent prognostic factors in lung adenocarcinoma patients (HR=0.43 and 3.18; p<0.02 and p<0.001, respectively).

### Relationship between CRBP-1 and other lung adenocarcinoma markers

CRBP-1^High^ correlated positively with EGFR^High^ expression (rho=0.38, p<0.01), and negatively with p53 expression (rho=−0.30, p<0.01). CRABP-2^High^ inversely correlated with bcl-2^High^ expression (rho=−0.25, p<0.05) and patients' age (rho=−0.28, p<0.05). An inverse correlation between EGFR and bcl2 expression (rho=−0.31, p<0.01) also existed. As reported in supplementary figure S1H, we compared keratins and nox4 expression according to CRBP-1 expression. In general, expression of keratins 1 and 5/6 was focal, whereas keratin 14 was not expressed. The percentages of tumor keratin 1 and 5/6 in CRBP-1^High^ was increased and almost double compared with CRBP-1^Low^ tumors. Instead the percentage of nox4 expression was similar. Screening for TK domain (exons 18-21; Supplementary figure S2A) documented that 19.8% of lung adenocarcinomas were EGFR-mutated, mostly in 19 and 21 exons, according to the literature [20,21]. We did not find mutations in 18 and 20 exons in our patients' cohort. A tendential correlation between EGFR-mutated status and CRBP-1^High^ expression was observed, although the association was not statistically significant (rho=0.21, p=0.15).

### CRBP-1 gene copy number and methylation in lung adenocarcinoma

As reported in supplementary figure S2B, 45.3% of lung adenocarcinomas showed 3-6, 42.85% two and 11.85% less than two copies of CRBP-1 gene. Increased CRBP-1 gene copy number associated with tumor CRBP-1^High^ status and dedifferentiation (rho=0.31; p<0.05). Since CRBP-1 expression was absent in a subset of tumors, we analyzed the methylation of the promoter region flanking the CRBP-1 gene (Supplementary figure S2C, D). Promoter methylation was present in 21.4% of adenocarcinomas and a correlation between CRBP-1^Low^ status and methylation gene expression was also documented (rho=−0.36, p<0.01).

### CRBP-1 transfection increased proliferation and retinoid sensitivity of A549 adenocarcinoma cells

**Figure 3:**
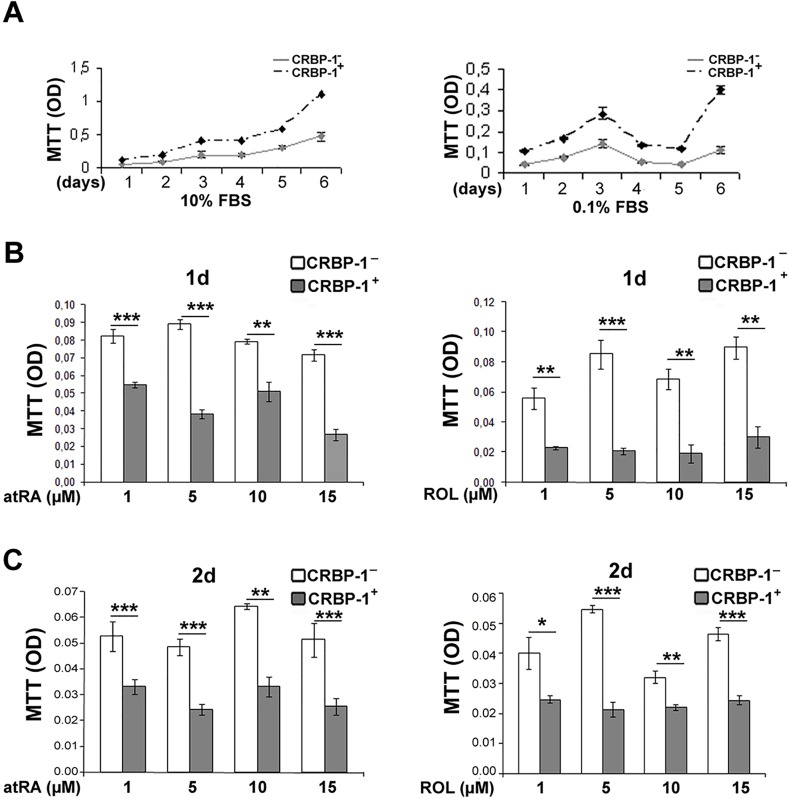
Viability and retinoid-related survival of CRBP-1-transfected A549 adenocarcinoma cells A, Serum-dependent of CRBP-1^+^ A549 cells growth is increased compared to CRBP-1^−^ cells. B-C, MTT assay shows reduced viability of CRBP-1^+^ compared to CRBP-1^−^ A549 cells after 1 and 2 days of *at*RA and ROL treatment in the presence of 0.1% FBS. Values are expressed as means ± SEM of three different experiments; *p < 0.05, **p < 0.005, ***p <<0.0005.

A mammalian CRBP-1-expressing vector was used to generate stable transfectant A549 lung adenocarcinoma cell lines. Wild A549 cells did not display appreciable CRBP-1 mRNA and protein levels (Supplementary figure S3A, B). After 6 days (figure 3A), MTT assay showed that CRBP-1^+^ grew more than CRBP-1^−^ A549 cells. Cell count gave similar results (not shown). At concentrations >1μM (figure 3B, C), *at*RA and ROL reduced viability more in CRBP-1+ than in CRBP-1− A549 cells. Finally, after 2 weeks of culture with 10% FBS, the ability of CRBP-1^+^ A549 cells to form colonies (66.0% ± 3.04%) slightly increased compared to CRBP-1^−^ cells (58.13% ± 2.92%), although the difference was not significant. Treatments with *at*RA and ROL reduced clonogenicity in both CRBP-1^+^ and CRBP-1^−^ A549 compared to control cultures (data not shown).

### CRBP-1 transfection influences differentiation and RAR/RXR signaling of A549 adenocarcinoma cells

**Figure 4:**
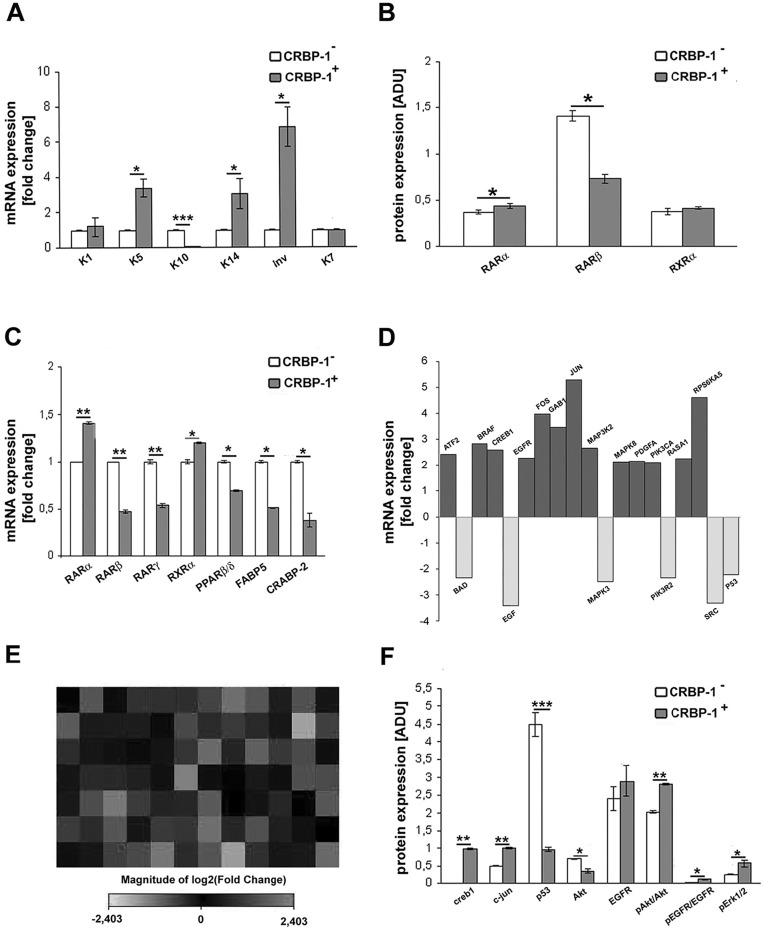
CRBP-1 transfection influences transcriptional pathways and differentiation of A549 adenocarcinoma cells A, bar graph after real-time PCR showing keratin (K) 1, 5, 14 and involucrin up-regulation, K10 down-regulation and unmodified K7 transcription level in CRBP-1^+^ compared to CRBP-1^−^ A549 cells. B, densitometric analysis of RARα, RARβ and RXRα protein expression by blot analysis. C, bar graph of RARs, RXRα, PPARβ/δ, FABP5 and CRABP-2 transcripts. D, bar graph and E, heat map of RT^2^ profiler^TM^ PCR assay of EGF/PDGF signaling-specific genes in CRBP-1^+^ A549 cells. Up-regulated and down-regulated genes are in dark grey and light grey, respectively. F, densitometric analysis of creb1, c-jun, p53, pAkt/Akt, pEGFR/EGFR and pErk1/2 protein expression by blots. Columns are means ± SEM of three different experiments. *p < 0.05, **p < 0.005 and ***p <0,001. Abbreviations: ADU, arbitrary densitometric units.

**Figure 5:**
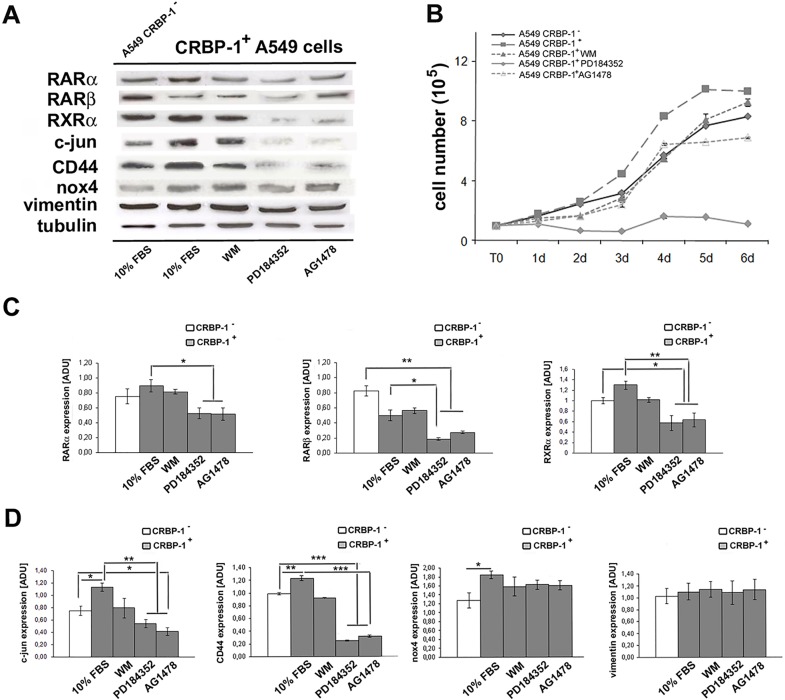
Effects of inhibition of AKT, MAPKK and EGFR activity in CRBP-1^+^ A549 cells A, Representative blots of RARs, RXRα, c-jun, CD44, nox4 and vimentin protein expression in CRBP-1^+^ A549 treated with inhibitors for 48 hours compared with CRBP-1^−^ A549 cells. B, cell growth graph of CRBP-1^−^ and CRBP-1^+^ A549 cells maintained with 10% of FBS and treated with wortmannin (WM), PD184352 and AG1478, as Akt, MAPKK and EGFR inhibitors up to 6 days. Densitometric analysis (C) RARs, RXRα, (D) c-jun, CD44, nox4 and vimentin protein expression. Membranes were reblotted with anti-tubulin to ensure equal loading. Results are mean values ± SEM of the three different experiments. *p < 0.05, **p < 0.005 and ***p <0,001. Abbreviations: ADU, arbitrary densitometric units.

In order to further investigate the effects on phenotype of CRBP-1 expression we performed proliferative, epithelial and epithelial to mesenchymal markers expression in A549 cells. Real-time PCR (figure 4A) showed the up-regulation of epithelial proliferative markers as keratin 1, 5 and 14 and involucrin and the down-regulation of keratin 10 transcripts in CRBP-1^+^ compared to CRBP-1^−^ A549 cells; keratin 7 level was unchanged. Keratin 5 and 14 up-regulation was confirmed by blots (data not shown). CRBP-1^+^ A549 cells also showed the strong up-regulation of nox4 and CD44 expression compared to CRBP-1^−^ A549 cells (figure 5A). The expression of other epithelial-to-mesenchymal transition markers such as vimentin, nestin, smad4, nanog, sox2, snail, TGFβ and MMP9 did not change (Supplementary figure S3C). We also evaluated the influence of CRBP-1 expression on retinoid signalling by blots and real time PCR. As reported in figure 4B and C, RARα and RARβ expression were up-regulated and down-regulated, respectively at both protein and transcriptional level in CRBP-1+ compared to CRBP-1− A549 cells; moreover, PPARβ/δ, FABP5 and CRABP-2 transcripts resulted down-regulated.

### CRBP-1 transfection associated with up-regulation growth and transcriptional gene levels in A549 adenocarcinoma cells

As reported in figure 4D and E, PCR array documented a series of up-regulated genes influencing cell growth and survival (BRAF, EGFR, GAB1, MAP3K2, MAPK8, PDGFA, PIK3CA, RASA1) and transcriptional activators (ATF2, CREB1, FOS, JUN, RPS6KA5) in CRBP-1^+^ compared to CRBP-1^−^ A549 cells. Instead, BAD, EGF, MAPK3, PIK3R2, SRC and P53 gene levels resulted down-regulated. Blot analysis (figure 4F) also documented the up-regulation of transcriptional factors creb1 and c-jun and of activity of survival and proliferative pathways, including pAkt, pEGFR and pErk1/2 in CRBP-1^+^ compared to CRBP-1^−^ A549 cells; instead p53 was down-regulated.

### Akt/Erk/EGFR inhibitors influence proliferative and epithelial-to-mesenchymal transition marker expression in CRBP-1^+^ A549 adenocarcinoma cells

In order to better identify those pathways regulated by CRBP-1 expression, we performed inhibition experiments. As reported in figure 5B, PD184352 MAPK inhibitor drastically reduced proliferation of CRBP-1^+^ A549 cells. Similar effects on proliferation were observed after wortmannin and AG1478 treatment. Moreover, as reported in figure 5C and D, PD184352 and AG1478 inhibitors strongly reduced RARβ, c-jun and CD44 expression in CRBP-1^+^ A549 cells. Also wortmannin-induced inhibition of pAKT reduced RARβ, RXRα, c-jun and CD44 expression to levels similar to CRBP-1^−^ A549 cells. All inhibitors did not change nox4 and vimentin protein expression.

## DISCUSSION

Our study results have shown that in a cohort of lung adenocarcinoma patients undergoing radical surgical treatment, tumor CRBP-1^High^ expression was associated with an unfavorable OS. In normal cells, CRBP-1 regulates intracellular retinol trafficking and bioconversion, so facilitating its biological functions [6]. In particular, retinol contributes to epithelial cell proliferation and differentiation. CRBP-1^High^ expression in lung adenocarcinomas was parallel to the increase of CRBP-1 gene copy number and also associated with increased tumor grade and keratin 1 and 5/6 expression, suggesting that CRBP-1^High^ expression reflects a more aggressive and dedifferentiated phenotype of adenocarcinoma cells. This was confirmed from the increased proliferation and dedifferentiation markers expression in CRBP-1- transfected A549 cells. Current paradigms retain that lung cancer arises from pluripotential stem cells capable of differentiation into one or several histological cell types, with the activation of genes recapitulating embryonic lung development. Lung adenocarcinoma has been associated with *de novo* gene expression of developmental terminal sac and alveolar stages, with a prevalence of genes influencing differentiation and signal transduction [22]. Our data are in line with the link between aberrant CRBPs expression and carcinogenesis described in non-lung districts, including laryngeal and hepatic cancer [10,12,13]. The same was reported in high-grade gliomas, where CRBP-1^High^ expression also associated with poor prognosis [23]. Aberrant CRBP-1 expression also occurred in non-epithelial malignant tumors, such as leiomyosarcomas [24]. Aberrant CRBP-1 expression was not univocal in non-lung epithelial malignancies. Loss of CRBP-1 expression has been reported in human dedifferentiated breast, endometrial and ovarian cancers [8,10,11,25]. It is likely that the prevalence of CRBP-1^High^ phenotype in lung adenocarcinoma facilitates intracellular retinoid level accumulation and trafficking supporting tumor cell proliferation and dedifferentiation in response to oncogenetic stimuli [6].

Over-expression of oncogenes or inactivation of tumor suppressor genes have been identified in a significant number of NSCLC patients [26]. Besides, clinical prognostic factors, such as stage, sex, and performance status, tumor molecular markers have been recognized to influence OS in NSCLC patients [27]. Our results strongly suggest that CRBP-1^High^ expression can be considered as an additional phenotypic marker of lung adenocarcinomas with a more aggressive clinical course. We also documented that RARα^High^, RARβ^High^ and CRABP-2^Low^ expression associates with reduced OS. We also documented that EGFR^High^ positively correlated with CRBP-1^High^ expression, strongly supporting an interaction between CRBP-1-mediated retinoid and EGFR pathways [28]. This finding is apparently in contrast with the overall reduced survival in patients with EGFR^Low^ expression. The literature contains conflicting data on the relationship between EGFR expression and survival in lung cancer. Variability and discrepancy of results may be due to heterogeneity of study population related to EGFR status at time of primary diagnosis, EGFR mutational status and/or chemotherapy [17]. CRBP-1-mediated increased transport of retinol to intracellular related enzymatic milieu is likely to amplify RAR-mediated transcriptional signals [29]. In epithelial cells, *at*RA and RAR-selective ligands specifically down-regulated EGFR-dependent activities and *at*RA regulated cell growth and differentiation [28]. Nevertheless, retinoids are known to control genes that do not contain classical RARE motifs, and to activate directly intracellular signaling molecules or transcription factors, such as Erk signaling components [30].

Our present data help to better understand the complex role and the contrasting opinions concerning the efficacy of retinoid-related in chemotherapeutic regimens in lung cancer patients. Retinoids are successfully used in preneoplastic or neoplastic skin diseases, head and neck cancer, neuroblastoma and cutaneous T-cell lymphoma. The use of retinoids for the treatment and prevention of lung cancer gave controversial results [31]. Preliminary evidence including preclinical and observational studies reported promising results, but these effects were not fully translated in human interventional settings. Encouraging results derived from efficacy of oral administration of high-dose vitamin A in reducing the number of primary tumors related to tobacco consumption and improving the disease-free interval in patients resected for stage I lung cancer [32]. Conversely, other trials found a significantly increased risk of lung cancer in current and former smokers. In particular, retinoids reduced tumor occurrence and mortality in non-smokers and were beneficial in former smokers, but increased the risk of lung cancer in smokers [16]. Our data demonstrating that OS was particularly reduced in smoker patients with CRBP-1^High^ tumor expression is highly suggestive. Although other studies are needed to clarify mechanisms responsible of higher incidence of lung cancers in smokers who received retinoids, it is likely that CRBP-1^High^ expression favors retinoid-induced proliferation of tumors cells. As matter of fact, CRBP-1 transfection increased proliferation, up-regulated RAR-α and down-regulated RARβ expression in A549 adenocarcinoma cells.

Gene array and blot documented that CRBP-1 transfection A549 cells induced the increase of transcription and activity of proliferative, transcriptional and dedifferentiative genes, including pEGFR/pAkt/pErk pathways, RARα, c-jun and CD44. CRBP-1 transfection effects were partially reverted by specific inhibitors of EGFR/Akt/Erk pathways. C-jun up-regulation has been reported to influence carcinogenesis and tumour progression in lung adenocarcinoma cells [33]. Our hypothesis is that CRBP-1 alone or homodimerized or heterodimerized with RARα or RARα/RXRα could interact and activate Akt and down-regulate related transcriptional, proliferative and dedifferentiative genes. This hypothesis is consistent with the previously reported finding that RARα over-expression increases activity and co-localizes with Akt at cell membrane level likely by interacting with PI3k [34]. Our *in vitro* results showed increased keratin 5, 14 and involucrin expression in CRBP-1^+^ A549 cells. Most of poorly differentiated adenocarcinomas express focally keratins 5, 6, 14 and 17 [35]. Coexpression of keratin 14, a basal cell marker of squamous and glandular epithelia, keratin 5 and involucrin are reported to represent a stem cell or progenitor cell phenotype in cancer cells [36]. Other experiences are needed to better clarify through with pathway CRBP-1 favors epithelial to mesenchymal transition in adenocarcinoma cells. In this light, we also described the increased expression of CD44 and nox4 in CRBP-1^+^ A549 cells. Increased CD44 expression was described to be associated with a poor outcome in lung adenocarcinoma patients and tumor progression [37].

In conclusion, in the present study we documented that CRBP-1^High^ expression in lung adenocarcinomas associates with a poor survival and increased tumor grade, likely influencing the activity of Akt/EGFR gene pathways. Further studies are needed to verify the possibility of CRBP-1-related therapeutic intervention aimed to reduce NSCLC progression for a more personalized chemotherapeutic regimens.

## PATIENTS AND METHODS

For the study purpose, 167 NSCLC patients who underwent surgical resection with histologic diagnosis of adenocarcinoma either at the Policlinic of Tor Vergata University of Rome and at the Santa Maria della Misericordia Hospital of Perugia, Italy, between 2003 and 2009 were included. Patients' written informed consent was obtained. The study was approved by the Local Ethics Committee. Tumor classification was in accordance with WHO criteria and the most diffuse immunohistochemical panel [38,39]. Tumor subtyping, grading and staging were in accordance with the international tumor-node-metastasis system (TNM) [39,40]. Criteria of exclusion were pre-operative radiation and/or chemotherapy and inadequate amount of tumor tissue for correct routinary processing and diagnosis (at least two tissue cores).

### Tissue microarray construction

For tissue microarray (TMA) construction, tissue samples from diagnostic biopsies and operative procedures were obtained from representative paraffin blocks maintaining patients' anonymity. All tumor slides were reviewed by light microscopy examination of Haematoxylin&Eosin (H&E)-stained sections. The most representative tumor areas were carefully selected and TMA constructed using positive and negative controls [8]. Serial 4 μm-thick sections were stained with H&E or employed for immunohistochemistry.

### Immunohistochemical study

For immunohistochemistry, sections were incubated with mouse monoclonal anti-human Ki-67 (clone 30-9), bcl2 (clone 124), p53 (clone DO-07), EGFR (clone 3C6), keratin 5/6 (clone D5/16B4) and keratin 14 (clone SP53) antibodies using an automatic immunostaining device (Ventana-Roche Diagnostics Milan, Italy) [41]. Serial sections were also incubated for 1 h with rabbit polyclonal anti-CRBP-1 (1:200; clone FL-135, Santa Cruz Biotechnology, Heidelberg, Germany), anti-CRABP-2 (1:300; Bethyl Laboratories, Montgomery, USA), anti-RARα (1:500; clone sc-551, Santa Cruz Biotechnology), anti-RARβ (cytoplasmic isoform β4; 1:100; clone ab53161 Abcam, Cambridge, UK) and anti-RXRα antibodies (1:500; clone sc-553, Santa Cruz Biotechnology), anti-keratin 1 (1:750; clone ab24643 Abcam), anti-Nox4 (1:500; H-300, Santa Cruz Biotechnology). Diaminobenzidine was used as final chromogen. Slides were also stained with a mouse monoclonal anti-CRBP-1 antibody (1:10, gifted from Dr ML Bochaton, University of Geneva, Switzerland), that gave similar results (not shown).

### CRBP-1 gene copy number variation and methylation

CRBP-1 gene copy number was analyzed by using real-time PCR and TaqMan Genotyping Master Mix (n. 4317355), with RNase control reagents (n. 4316844) and CRBP-1 (Hs00443703, Applied Biosystem, Foster City, CA, USA) as probe. PCR amplification was performed in an ABI PRISM 7500 (Applied Biosystem) according to manufacturer's instructions. Data analysis was performed using the manufacturer's integrated web-based software package. Genomic DNA, methylation specific PCR and copy number assay were performed as reported [8]. Briefly, DNA was isolated by using a FFPE tissue kit (Qiagen, Hilden, Germany) and PCR carried out using AmpliTaq Gold DNA polymerase (Applied Biosystem) and specific primers, as reported [8].

### EGFR mutational status

EGFR mutational status was analized by pyrosequencing [42]. The TK domain of the EGFR coding sequence involving exons 18-21 was amplified by using the EGFR TKI Response PQ (Diatech Pharmacogenetics, Jesi AN, Italy) and then sequenced using PyroMark Q24 instrument (Qiagen).

### Cell transfection

Human A549 adenocarcinoma cells (Sigma-Aldrich, St. Louis, USA) maintained in RPMI 1640 (Lonza Bio Pharma AG, Switzerland) were transfected ([43]) by using a vector pTargeT Mammalian expression system carrying the whole sequence of CRBP-1 gene (NM_002899) and the gene for the resistance to G418 (CRBP-1^+^, Promega, Italy), or the G418-resistance gene alone (CRBP-1-). After 20 days, stable transfected clones were collected and tested by PCR and western blot. The correct plasmid sequence was confirmed by Sanger sequencing. Experimental procedures were repeated by using two different transfected clones, which gave similar results (not shown).

### Cell growth and viability

For proliferation studies, overnight serum-starved cells were treated with different concentrations of *at*RA and ROL (Sigma-Aldrich) in 0.1% FBS up to 6 days. For cell viability, 3-(4,5 dimethylthiazol-2-yl)-2,5diphenyl-tetrazolimbromide assay (MTT; Sigma-Aldrich) was carried out in triplicate ([44]. In some instances, CRBP-1^+^ cells were pre-treated with selective EGFR (AG1478; Sigma-Aldrich; 10 μM), phosphatidylinositol 3-kinase/ AKT (Wortmannin; Sigma-Aldrich; 10 μM) and the mitogen-activated protein kinase kinase (MAPKK, PD184352; Sigma-Aldrich; 2μM) inhibitors.

### Clonogenic assay

For the clonogenic assay ([45], cells were seeded and treated with different concentrations of *at*Ra and ROL. Colonies arising from survival cells were fixed and stained with 1% methylene blue (Sigma-Aldrich) in 0.1% methanol and their percentages as plating efficiency (PE) calculated.

### Western blot analysis

After isolation, content determination and electrophoresis, proteins were elettroblotted [46] and incubated with a polyclonal rabbit anti-CRBP-1, anti-Creb1, anti-CD44, anti-c-Jun, anti-Nox4, anti-p53, anti-RXRα, anti-RARα (Santa Cruz Biotechnology), anti-RARβ, (Abcam), anti-phosphorylated v-akt murine Jesi AN, Italy) and then sequenced using PyroMark Q24 thymoma viral oncogene homolog (pAkt Ser^473^), anti-AKT (pan), anti-phosphorylated extracellular-signal-regulated kinases (pErk1/2), anti-phosphorylated epidermal growth factor receptor (anti-EGFR Thr669), anti-EGFR antibody (Cell Signaling Technology, Danvers, MA, USA), anti-keratin 5 (clone H-40, Santa Cruz Biotechnology), mouse anti-vimentin (clone J144, Abcam), anti-keratin 14 (LL001, Santa Cruz Biotechnology) and anti-total tubulin antibody (Sigma-Aldrich). Revelation and densitometric blot analysis were performed in three independent experiments and Akt and EGFR activity expressed as phospho/total protein ratio [47].

### Gene expression analysis

Total RNA was extracted [48], reverse-transcribed and a commercially available RT profiler PCR array of 86 genes human EGF/PDGF signaling (PAHS-040Z, Qiagen) and Real time PCR performed according to the manufacturer' instruction and primers listed in supplementary table S1; Δ2-microglobulin, Δ-actin and glyceraldehyde-3-phosphate dehydrogenase (GAPDH) were used as housekeeping genes. Data analysis was performed by using the integrated web-based software package using ∆∆C_t_ fold-change calculation (Qiagen) in triplicate experiments.

### Semiquantitative and statistical analysis

CRBP-1, CRABP-2, nox4 and keratin expression was estimated at 400X magnification by two of the Authors by using the following semiquantitative grading system: absent (0), weakly positive <50% (0.5), moderately positive <50% or weakly positive >50% (1), strongly positive <50% or moderately positive >50% (2), strongly positive >50% (3), as reported [25]. Inter-observer reproducibility was >95%. Tumors with 0-0.5 CRBP-1 and CRABP-2 score were arbitrarily grouped as “Low” and those with 1-3 score as “High”. For p53, Ki-67, bcl-2 and RAR/RXR, tumors were arbitrarily considered as “Low” when expression was less than 20%. Continuous moderate or marked membranous staining in >50% of cells was required for the definition of EGFR^High^ expression [49]. For each case, the ratio of the score and the number of analyzed fields was calculated. Results were analyzed by means of Student's *t* test. Univariate analysis of relationship among CRBP-1 and clinicopathological variables was performed by using χ^2^ test, whereas correlations between CRBP-1 and the other biomarkers by Spearman's rank correlation test. Survival curves were analyzed by Kaplan-Meier method and significant differences between subgroups were calculated by the *log-rank test*. Independent prognostic factors were identified by multivariate analysis using Cox proportional hazards model. Only factors showing prognostic significance in univariate analysis were adopted in multivariate analysis. Differences were considered statistically significant for value of *p*<0.05. SPSS 16 software program (Spss inc. Chicago, IL, USA) was used for statistical analysis.

## SUPPLEMENTARY MATERIAL FIGURES AND TABLE


