# Supplementary material for: High expression of cellular retinol binding protein-1 in lung adenocarcinoma is associated with poor prognosis
Source: Genes Cancer. 2015 Nov;6(11-12):490–502. doi: 10.18632/genesandcancer.89 (PMC4701228; doi:10.18632/genesandcancer.89)
Supplement: Supplementary file 1 [file ganc-06-490-s001.pdf]

High expression of cellular retinol binding protein-1 in lung adenocarcinoma is associated with poor prognosis

Supplementary Material

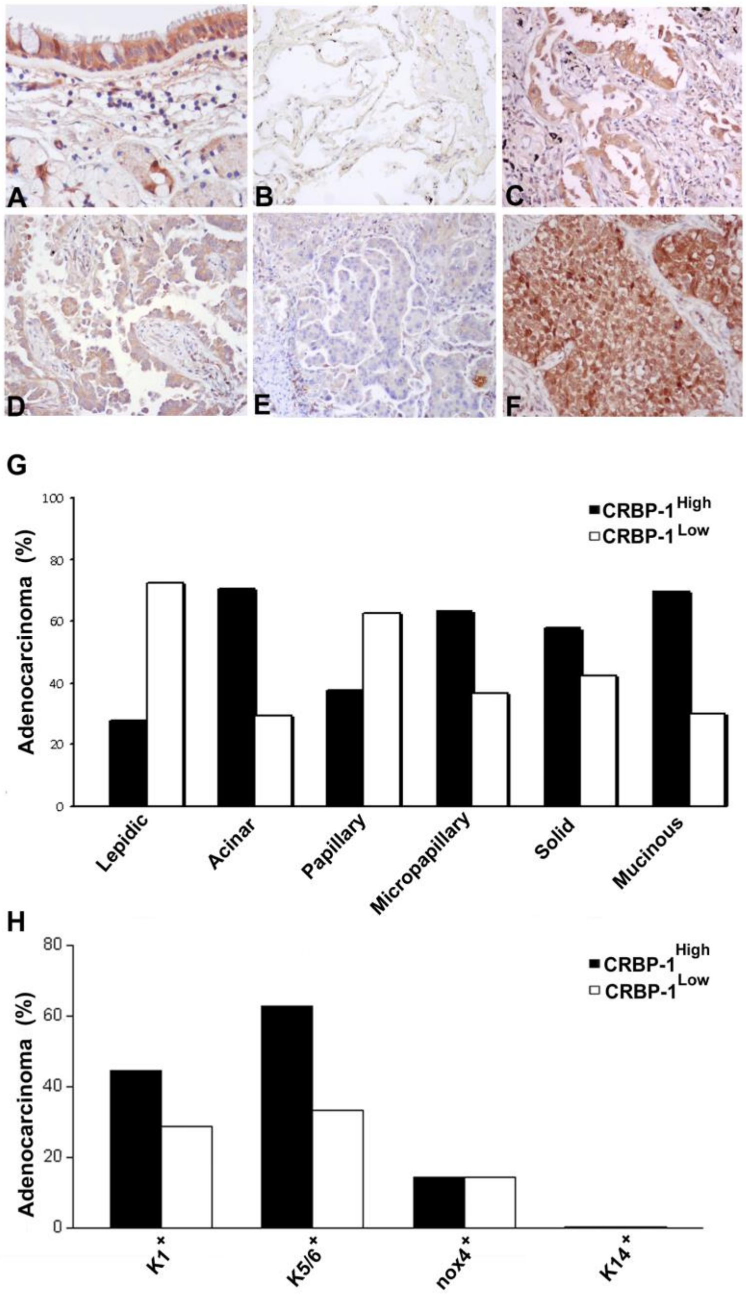

Supplementary Figure S1. Representative CRBP-1 immunostaining of non-neoplastic lung tissue and of major histological patterns of invasive lung adenocarcinomas and percentages of expression. **A**, normal bronchial epithelium. **B,** normal lung. **C,** acinar. **D,** papillary, with evident tumor cells growing on the surface of fibrovascular cores. **E,** micropapillary, with small papillary clusters

growing within air spaces and **F,** solid patter. **G,** bar graph showing the percentage of CRBP-1^{High} and CRBP-1^{Low} expression adenocarcinomas according to the histological subtype (1). Original magnification: *A*, x400; *B*-*F*, x125. **H,** bar graph showing the percentages of tumor keratin (K) 1, 5/6, 14 and nox4 expression in CRBP-1^{High} and CRBP-1^{Low} adenocarcinomas.

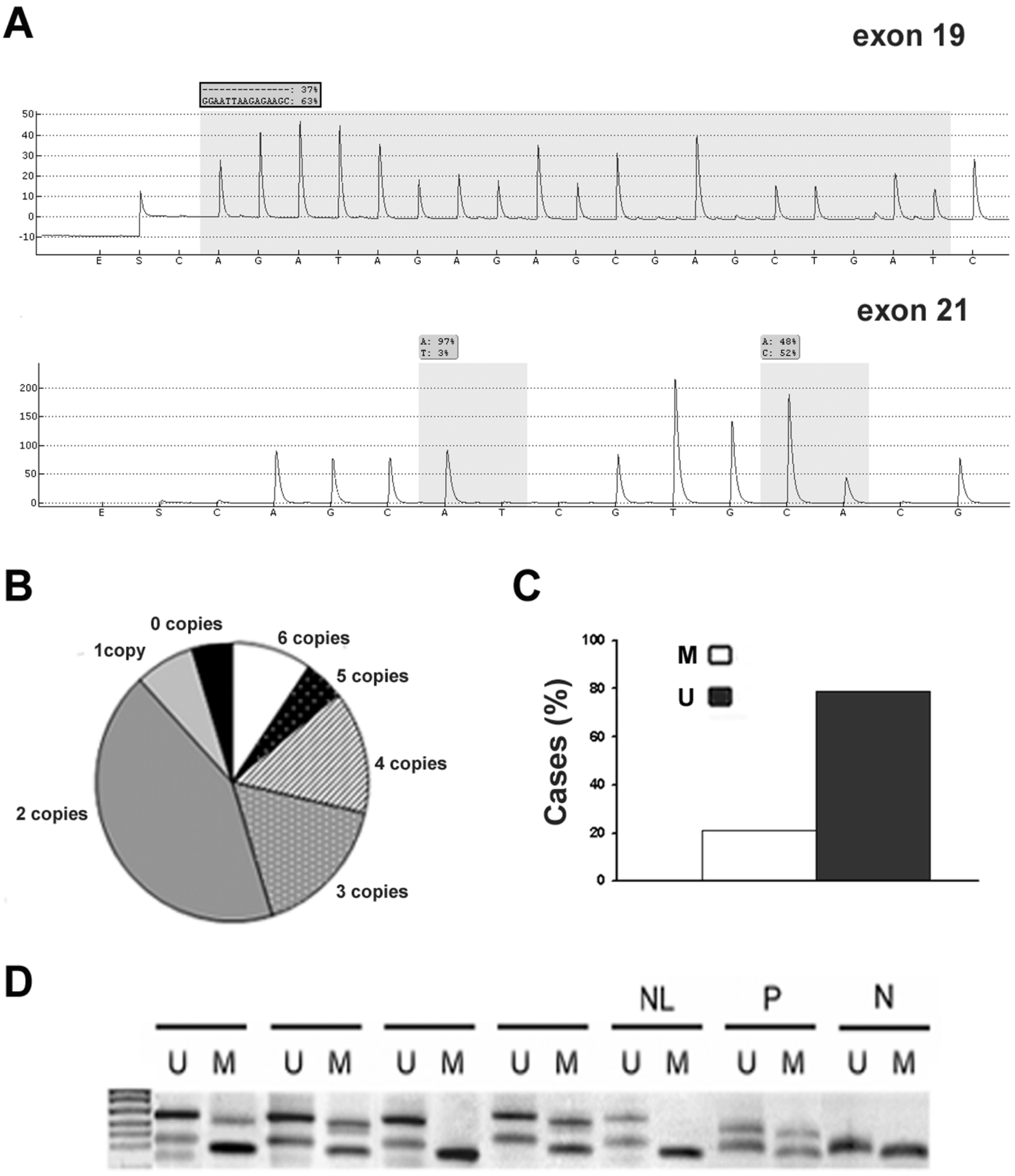

Supplementary Figure S2. Representative EGFR mutational status, CRBP-1 gene copy number gain and promoter methylation in lung adenocarcinomas. **A,** representative pyrograms of mutated 19 and 21 EGFR exons in lung adenocarcinoma. **B,** pie chart of CRBP-1 gene copy number gain in lung adenocarcinoma tissues. **C,** bar graph of percentages of methylated and unmethylated CRBP-1 lung adenocarcinomas. **D,** representative lanes of the methylation *status*. M and U lanes correspond to specific amplified products of methylated and unmethylated DNA, respectively. Methylated samples are marked with asterisks. Normal lung (NL) as negative and DNA (Qiagen, “P”) as positive control were used to ensure primer specificity; “N” no template control.

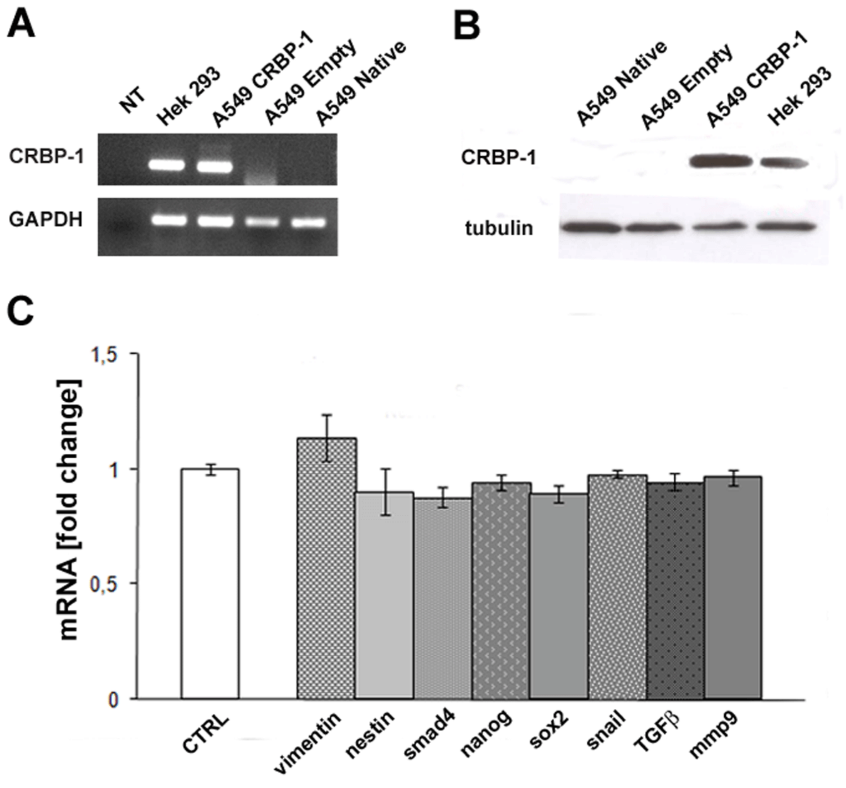

Supplementary Figure S3. CRBP-1 transcript and protein in wild, empty vector and CRBP-1-transfected A549 adenocarcinoma cells. **A,** RT-PCR shows evident CRBP-1 transcript in transfected A549 cells. Glyceraldehyde 3-phosphate dehydrogenase (GAPDH) is used as housekeeping gene. **B,** representative blots of protein expression in CRBP-1-transfected A549 lung adenocarcinoma cells. Membranes were reblotted with anti-tubulin to ensure equal loading, and Hek-293 cells as positive control. **C,** transcript levels of epithelial-to-mesenchimal transition genes in CRBP-1^{+} compared to CRBP-1^{-} A549 cells (CTRL).

Suppplementary Table S1. Primers Sequences

Gene	Primer Sequence	Accession Number
GAPDH	Sense: 5’-ACGGATTTGGTCGTATTGG-3’<br>Antisense: 5’-GATTTTGGAGGGATCTCGC-3’	NM_002046
ß2-microglobulin	Sense: 5’-GATTCAGGTTTACTCACGTC-3’<br>Antisense: 5’-GTTCACACGGCAGGCATACT-3’	NM_004048
CRBP-1	Sense: 5’-CGCTTGTGG CCAAACTGGCTC-3’<br>Antisense: 5’-ACACATCCTGCTGATTGGTTGG- 3’	NM_002899.3
CRABP-2	Sense: 5’-GCCCAACTTCTCTGGCAACTG-3’<br>Antisense: 5’-GTCTGCTCCTCAAACTCCTCCC-3’	NM_001878.3
RARα	Sense 5’-GGTCGGCGATGGTGAGGGT-3’<br>Antisense 5’-TGGGCAAATACACTACGACAACAG-3’	NM_001145302.2
RARß	Sense: 5’-GCAGAGCGTGTAATTACCTTGAA-3’<br>Antisense: 5’-GTGAGATGCTAGGACTGTGCTCT-3’	NM_016152.3
RARγ	Sense: 5’-CTGCCAGTACTGCCGGCTAC-3’<br>Antisense: 5’-TCTGCACTGGAGTTCGTGGTATACT-3’	NM_000966.5
RXRα	Sense: 5’-TCCTCCTCCCACCGCTCCATC-3’<br>Antisense: 5’-CAGCTCCGTCTTGTCCATCTG-3’	NM_002957.4
FABP-5	Sense: 5’-CCTGTCCAAAGTGATGATGG-3’<br>Antisense: 5’-CAGCATCAGGAGTGGGATG-3’	NM_001444.2
PPARß/δ	Sense: 5’-ATGGAGCAGCCACAGGAGGAAGCC-3’<br>Antisense: 5’-GCATGAGGCCCCGTCACAGC-3’	NM_006238.4
K1	Sense: 5’-ATTTCTGAGCTGAATCGTGTGATC-3’<br>Antisense: 5’-CTTGGCATCCTTGAGGGCATT-3’	NM_006121.3
K10	Sense: 5’-TGATGTGAATGTGGAAATGAATGC-3’<br>Antisense: 5’-GTAGTCAGTTCCTTGCTCTTTTCA-3’	NM_000421.3
K5	Sense: 5’-CAGCGTCAAATTTGTCTCCAC-3’<br>Antisense: 5’-TTGGTCTAGACTACTCTCCAG-3’	NM_000424.3
K7	Sense: 5’-TGAATGATGAGATCAACTTCCTCAG-3’<br>Antisense: 5’-TGTCGGAGATCTGGGACTGC-3’	NM_005556.3
K14	Sense: 5’-CCAGTTCTCCTCTGGATCGCAG-3’<br>Antisense: 5’-GATCTTCCAGTGGGATCTGTGTCCA-3’	NM_000526.4
Involucrin	Sense: 5’-GGGTGGTTATTTATGTTTGGGTGG-3’<br>Antisense: 5’- GCCAGGTCCAAGACATTCAAC-3’	NM_005547.2
Nestin	Sense: 5'-GCGGTGGCTCCAAGACTTC-3'<br>Antisense: 5'-ACTGGGAGCAAAGATCCAAGAC-3'	NM_006617.1
SMAD-4	Sense: 5'-TTCTGGAGGAGATCGCTTTTG-3'<br>Antisense: 5'-TTGCCTATGTGCAACCTTGCT-3'	NM_005359.5
NANOG	Sense: 5'-GCAATGGTGTGACGCAGAAG-3'<br>Antisense: 5'-AGGTTCCCAGTCGGGTTCA-3'	NM_024865.3
SOX-2	Sense: 5'-CCGTTCATCGACGAGGCTAA-3'<br>Antisense: 5'-TCATGAGCGTCTTGGTTTTCC-3'	NM_003106.3
Vimentin	Sense: 5'-TCCAAGTTTGCTGACCTCTCTG-3'<br>Antisense: 5'-CAGTGGACTCCTGCTTTGCC-3'	NM_003380.3
SNAIL	Sense: 5'-TGCATATTCGGACCCACACA-3'<br>Antisense: 5'-TGTTGCAGGAGGGCAAGAA-3'	NM_005985.3
TGF-β	Sense: 5'-GGACACCAACTATTGCTTCAG-3'<br>Antisense: 5'-TCCAGGCTCCAAATGTAGG-3'	NM_000660.5
MMP-2	Sense: 5'-TGGCAAGTACGGCTTCTGTC-3'<br>Antisense: 5'-TGGCAAGTACGGCTTCTGTC-3'	NM_004530
MMP-9	Sense: 5'-TGCGCTACCACCTCGAACTT-3'<br>Antisense: 5'-GATGCCATTGACGTCGTCCT-3'	NM_004994

References

1. Kadota K, Suzuki K, Kachala SS, Zabor EC, Sima CS, Moreira AL, et al. A grading system combining architectural features and mitotic count predicts recurrence in stage I lung adenocarcinoma. Mod Pathol 2012;25:1117-1127.
